# Evaluating local vegetation cover as a risk factor for malaria transmission: a new analytical approach using ImageJ

**DOI:** 10.1186/1475-2875-13-94

**Published:** 2014-03-13

**Authors:** Emily E Ricotta, Steven A Frese, Cornelius Choobwe, Thomas A Louis, Clive J Shiff

**Affiliations:** 1 W. Harry Feinstone Department of Molecular Microbiology and Immunology, Johns Hopkins Bloomberg School of Public Health, Baltimore, Maryland, USA; 2 Department of Viticulture and Enology, University of California, Davis, CA, USA; 3 Malaria Institute at Macha, Johns Hopkins Malaria Research Institute, Macha, Zambia; 4 Department of Biostatistics, Johns Hopkins Bloomberg School of Public Health, Baltimore, MD, USA

**Keywords:** Malaria, Vegetation, Vector behavior, Satellite imagery, ImageJ

## Abstract

**Background:**

In places where malaria transmission is unstable or is transmitted under hypoendemic conditions, there are periods where limited foci of cases still occur and people become infected. These residual “hot spots” are likely reservoirs of the parasite population and so are fundamental to the seasonal spread and decline of malaria. It is, therefore, important to understand the ecological conditions that permit vector mosquitoes to survive and forage in these specific areas. Features such as local waterways and vegetation, as well as local ecology, particularly nocturnal temperature, humidity, and vegetative sustainability, are important for modeling local mosquito behavior. Vegetation around a homestead likely provides refuge for outdoor resting of these insects and may be a risk factor for malaria transmission. Analysis of this vegetation can be done using satellite information and mapping programs, such as Google Earth, but manual quantification is difficult and can be tedious and subjective. A more objective method is required.

**Methods:**

Vegetation cover in the environment is reasonably static, particularly in and around homesteads. In order to evaluate and enumerate such information, ImageJ, an image processing software, was used to analyse Google Earth satellite imagery. The number of plants, total amount of vegetation around a homestead and its percentage of the total area were calculated and related to homesteads where cases of malaria were recorded.

**Results:**

Preliminary results were obtained from a series of field trials carried out in South East Zambia in the Choma and Namwala districts from a base at the Macha District Hospital.

**Conclusions:**

This technique is objective, clear and simple to manipulate and has potential application to determine the role that vegetation proximal to houses may play in affecting mosquito behaviour, foraging and subsequent malaria incidence.

## Background

Mosquito survival and foraging are driving factors in the overall epidemiology of malaria. At times when conditions are harsh and dry, survival of the vectors is limited and local foci of transmission persist because of shrub vegetation that provides mosquitoes with resting sites and refuges [1]. Where malaria transmission is unstable or seasonally restricted by dry, hot seasonal weather, predicting or defining conditions that will sustain pockets of vectors will be of considerable epidemiological importance [2]. On a local scale, foci are crucial for the detection and elimination of small, isolated transmission reservoirs. These “hot spots” are particularly important during periods of low transmission, as they are a primary source of infection for resurgent mosquito populations [3,4]. As the existence and stability of these foci are a function of climate conditions that enable the mosquitoes to forage, features of the landscape and ecology in a region are likely to play a major role in malaria transmission dynamics by affecting mosquito development, survival, and foraging behavior [5]. Such features include land surface temperature, water bodies, altitude, rainfall, and vegetation [6]. It is important to keep in mind how mosquito behaviour is affected by environmental conditions when evaluating the biological significance of these features and the role they play in the risk of malaria transmission.

However, while these ecological factors are important to know, they are not fine-scale enough to determine the location of site-specific transmission. Instead, by looking at local waterways and vegetation in addition to considering local ecology (specifically nocturnal temperature, humidity, and vegetative sustainability), local mosquito behavior can be predicted [7,8]. For example, as shrubs and smaller plants can create or alter the microclimate in which mosquitoes can rest outside of the home, vegetation around a homestead is likely to be an important determinant of malaria transmission [9]. This is particularly so during the dry season as resting sites can be sparse and the amount of available vegetation could affect mosquito survivorship and dispersal away from the home, and therefore affect malaria risk in a particular area. Such fine-tuned environmental data can be obtained via satellites, and using aerially-obtained environmental data is an excellent way to evaluate the spatial element of malaria transmission. The choice of satellite data depends on the study design and questions, as well as resource availability.

Current methods for assessing vegetation cover using remote sensing require complex calculations to make the data interpretable for use in epidemiological analyses [6]. The primary method for evaluation of vegetation cover is the normalized difference vegetation index (NDVI) [8]. This is the ratio of near infrared and red spectral bands obtained via SPOT imagery, which can quantify green leaf vegetation that can be mapped and used in statistical models [10]. Here, a method for evaluating vegetation without requiring complex satellite data and intricate calculations was devised. However observing and in some way measuring the extent of vegetation cover from satellite maps is somewhat subjective and difficult to interpret, it can be done more objectively and consistently using a computer program.

The software used for this analysis was ImageJ, a public domain Java image processing program created by the National Institutes of Health [11]. It has image processing capabilities and is used for a wide variety of applications. By measuring pixel values and making simple calculations about pixel size, number, area covered, etc., ImageJ can be used to measure distance between spots, create plots for graphic visualization, and transform images through automated macros, thus reducing measurement bias. In this study, ImageJ was used to analyse freely-available Google Earth images of malaria-endemic locations to identify potential risk factors associated with vegetation cover.

## Methods

### Household selection

**Figure 1:**
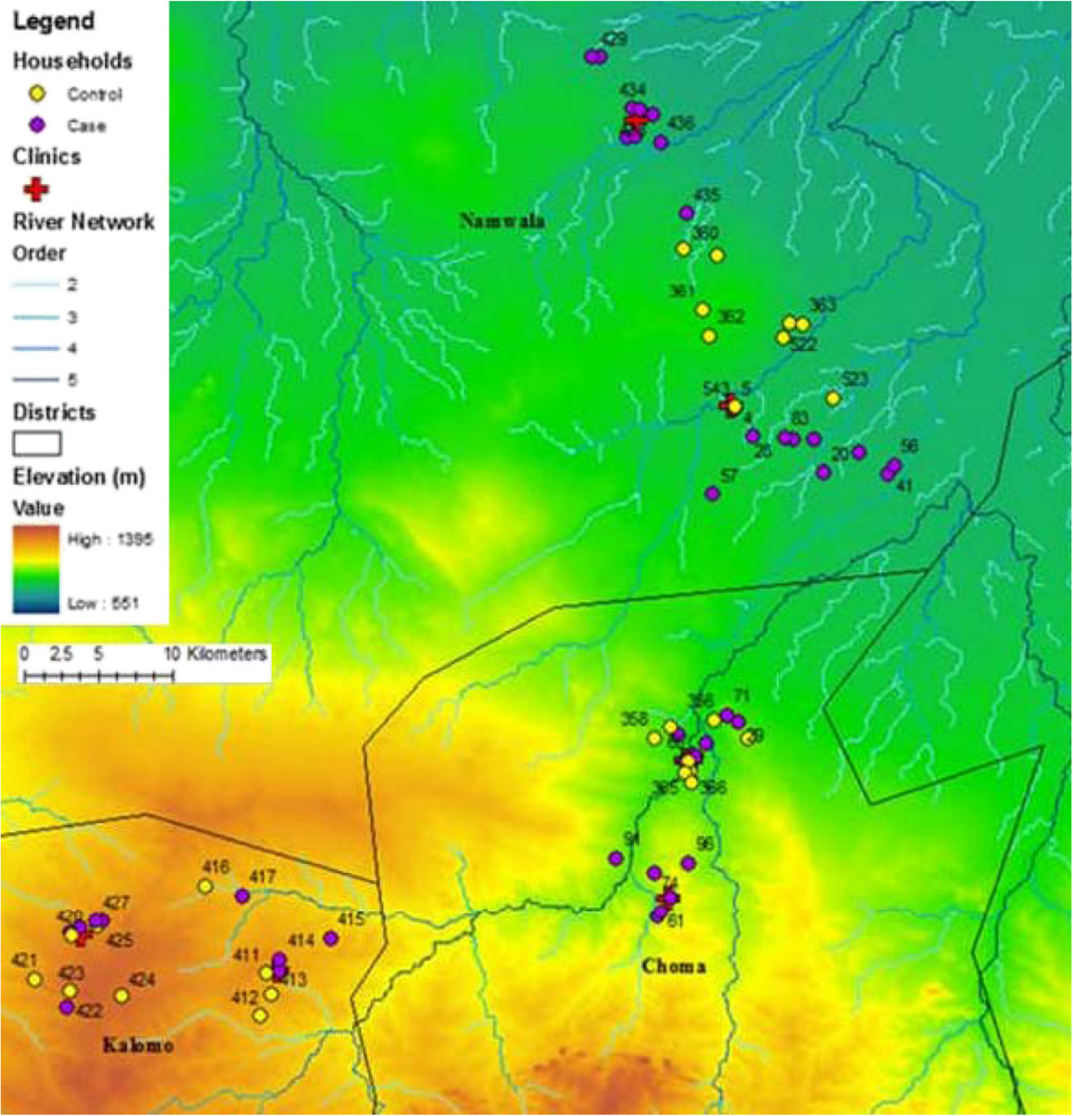
**Map of study area in Macha, Zambia.** This map shows elevation (m) and the river network derived from a digital elevation model. Two clinics for each district (Namwala, Choma, and Kalomo) were selected and households around each were sampled. Controls were randomly selected from clinic ledgers from houses with a non-malaria diagnosis.

Households for this study were located in Macha, Southern Province, Zambia (16° 26′ 0″ South, 26° 47′ 0″ East). The study site consists of an approximately 5,000 km^2^ area of the Kalomo, Choma, and Namwala districts in the Macha region (Figure 1). Beginning in August 2008 and occurring annually through 2012, all RDT-positive malaria cases detected in the region’s rural health centers were recorded. There are few villages in the area; people generally live on farms in clusters of houses, or homesteads with 8–30 people per homestead. Experimental homesteads were selected from locations where an RDT-positive case resided. These homesteads were visited and GPS coordinates were obtained, as described previously [12]. Controls were selected among homesteads visited in the same time period that were occupied by either RDT-negative residents or who had been selected from clinic registers as having a non-malaria diagnosis. There were a total of 122 homesteads available for analysis in this study.

### Mapping

**Figure 2:**
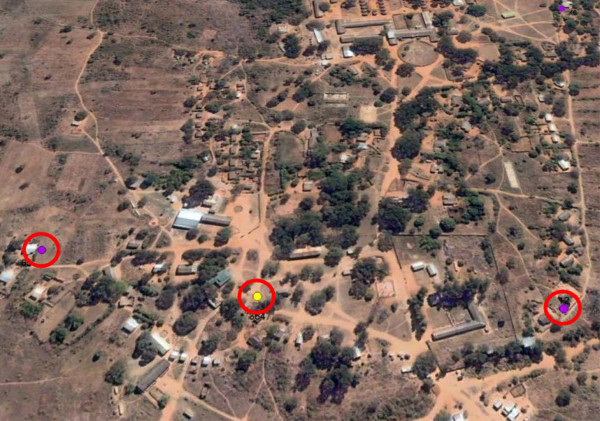
**Homesteads mapped on satellite imagery.** Images from June 2007 were obtained from DigitalEarth’s QuickBird satellite at a resolution between 2.4 m – 10 m per pixel. Data points were geo-referenced with UTM zone 35S, WGS 1984. Three homesteads can be seen grouped around Mapanza clinic. This image is representative of those used in the analysis from Google Earth.

Homesteads were mapped using ArcGIS (ESRI 2009, ArcGIS Desktop: Release 9.3 Redlands, CA: Environmental Systems Research Institute) and overlaid onto Google Earth imagery of the Macha region for a high-resolution view of the area. These images were used to evaluate waterways, local vegetation, and other topographic features (Figure 2). The most recent imagery obtainable was from June 2007, at a resolution between 2.4 m – 10 m per pixel. The majority of images are provided to Google via the QuickBird satellite owned by DigitalEarth.

### ImageJ

ImageJ is an image analysis software program, which has found wide use in a variety of fields. The program is Java-based and will run on all computer platforms, is freely available and easy to use, and can be customized to support a variety of new functions. Further, it can easily handle large data sets, extending its utility to applications which require uniform analysis over many repetitions. Here, these features were utilized to analyse satellite imagery of homesteads for surrounding vegetation.

### ImageJ protocol development and image analysis

Vegetation in Zambia differs by season but consists primarily of grasses, short shrubbery, and patches of trees. In addition, much of the area surrounding homesteads are croplands. Of the different types of vegetation present, short grasses are least visible on satellite data; however, as dry grass provides little shelter for vector mosquitoes, these areas were not included in this analysis. Of the remaining vegetation, short trees and bushes provide more suitable shelter for post-feeding resting mosquitoes and these also produce the largest signal difference in satellite imagery.

**Figure 3:**
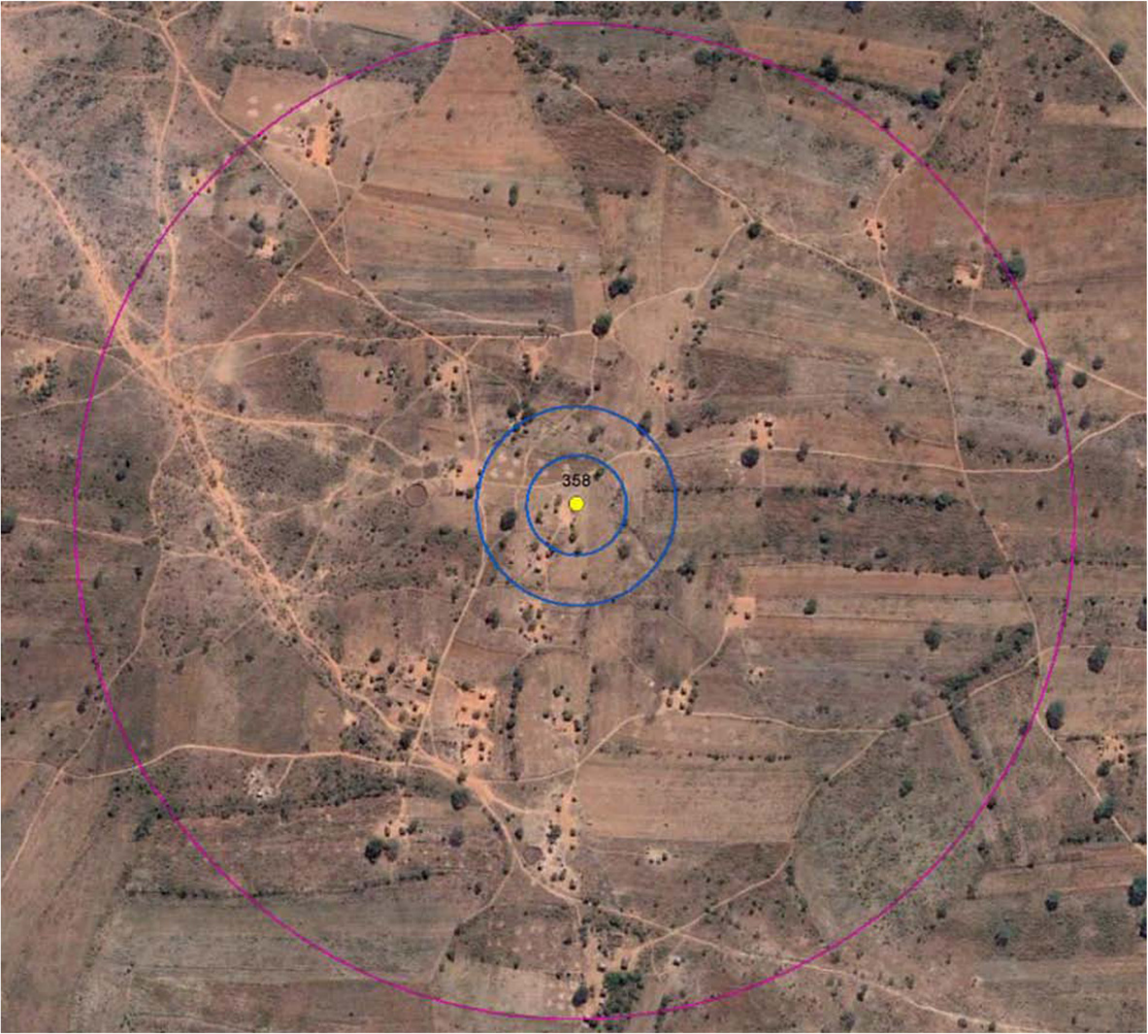
**Homestead buffers for vector dispersal.** DigitalEarth’s QuickBird image from June 2007, 2.4 m/pixel. Buffers were created at 50, 100, and 500 m to simulate vector dispersal using ArcGIS 9.3. This image is representative of those used in the analysis from Google Earth.

To simulate vector dispersal distance [13], buffers were created at 50, 100, and 500 m around each homestead using ArcGIS (Figure 3). Within each buffer, number of plants was recorded using ImageJ. To do this, images obtained from Google Earth of the Macha region were adjusted for brightness and contrast to maximize the difference between vegetation and ground surface or short grasses. An area scale was created for each image resolution group based on buffer radius (50, 100, or 500 m). This scale was used to calculate the pixel/meter ratio for the image and ultimately the area of vegetation surrounding each homestead, as there are more pixels/meter in the high resolution images. Images were converted to an 8bit black and white image, the threshold was adjusted as described below, noise was removed using ImageJ’s despeckle filter, and vegetative area was analysed in the buffer zones around each homestead using the Analyze Particles function in ImageJ (Additional file 1: Figure S1, Additional file 2: Figure S2, Additional file 3: Figure S3, Additional file 4: Figure S4, Additional file 5: Figure S5). The process was automated using ImageJ's macro feature so that each stack of images could be analysed under identical parameters. An accuracy assessment was attempted by counting the number of plants around each homestead by hand, but due to the unreliability which resulted in high variation in number of plants (indeed the reason this ImageJ technique was developed), this method of assessment was abandoned. To validate this process, the same set of households (100 m buffer) used in the analysis were run multiple times to determine its reproducibility. After analysis was run, the results were exported into Microsoft Excel for use in statistical models.

### Threshold selection

Threshold values were analysed in five unit increments to determine the optimum threshold values for area calculation. Representative images were adjusted similarly for brightness and contrast, and thresholds were set (0–60, 0–65, 0–70, etc.) and analysed, as above. The results were compared across threshold values and an optimum threshold value was chosen based on a combination of these results and a visual selection.

### Statistical analysis

All relations were analysed by logistic regression using R version 3.0.0 (2013-04-03) -- “Masked Marvel” (The R Foundation for Statistical Computing) to estimate odds ratios, with malaria case status as the outcome of interest (case = 1, control = 0). 2.4 m/pixel and 10 m/pixel resolution images were pooled to increase the power of the study. Each analysis was repeated for images at radii of 50, 100, and 500 m. Variables were count (number of plants), average size of plants, fraction of the area surrounding the homestead, and total area covered by plants. Quartiles of each variable were also analysed.

### Ethical clearance

The work was carried out under the approval of the Johns Hopkins Bloomberg School of Public Health IRB no: 00002290. All data was de-identified by MIAM staff before transmission to Johns Hopkins. No patient data was retained other than the GPS coordinates of the household, which was approved by this IRB, and therefore no written consent was obtained.

## Results and discussion

Of the 122 homesteads with available satellite imagery, 79 had usable images (36 case homesteads and 43 control homesteads). Only households that had usable imagery at all three buffer distances were included in the analysis. Reasons for exclusion include poor quality imagery, images combining two different resolutions, and households covered by clouds at the time the image was captured. These are common concerns when evaluating any satellite imagery, especially those from Google Earth, as satellites only pass over locations at certain times and data are not routinely updated. Of note, these images were from June 2007, while homesteads were from 2008–2012. However, as the landscape has not significantly changed in this area (and in fact all homesteads were able to be located on the images), the time discrepancy is not believed to be an issue. This would have to be assessed in each location on a study-by-study basis.

The usable images were processed with ImageJ with the threshold set at a minimum of 75, default B&W, dark background. This threshold was chosen because after narrowing the range of thresholds to between 60 and 100, visually, 75 captured the plants most accurately (Additional file 6: Figure S6, Additional file 7: Figure S7, Additional file 8: Figure S8, Additional file 9: Figure S9, Additional file 10: Figure S10). When evaluating the data output from all of the threshold values, the changes were not substantially different. In one example, thresholds 60–75 all produced a count of 927 plants, threshold 80 had 937 plants, and 85–100 had 942. Whether this difference is great enough to significantly affect statistical analyses will require further research. After the threshold was selected, the Analyze Particles settings were set to pixel size 0-infinity and circularity 0.00-1.00. Values for each of the four variables were obtained for all 79 houses and were exported to Excel for statistical analysis. Results from the statistical analyses can be seen in Tables 1 and 2.

**Table 1:** Odds ratios of variables when included in regression model as continuous

**Average size**			
**Buffer (m**^**2**^**)**	**OR (95% CI)**	**SE**	**p-value**
50	1.02 (0.99-1.05)	0.014	0.152
100	0.99 (0.97 – 1.02)	0.012	0.799
500	0.99 (0.99 – 1.01)	0.005	0.502
**Area fraction**			
50	1.01 (0.96 – 1.06)	0.026	0.662
100	0.98 (0.93 – 1.04)	0.028	0.517
500	0.97 (0.91 – 1.03)	0.031	0.388
**Total area**			
50	1.00 (0.99 – 1.00)	0.0003	0.313
100	0.99 (0.99 – 1.00)	0.00009	0.513
500	0.99 (0.99 – 1.00)	0.000004	0.437
**Count**			
50	1.00 (0.99 – 1.01)	0.041	0.830
100	1.00 (0.99 – 1.01)	0.004	0.943
500	0.99 (0.99 – 1.00)	0.0008	0.548

**Table 2:** Odds ratios of variables when included in regression models as categorical quartiles

**Average size**				
**Buffer (m**^**2**^**)**	**Quantile**	**OR (95% CI)**	**SE**	**p-value**
50	(17.6,26.1]	1.34 (0.34 – 4.56)	0.65	0.744
	(26.1,38.2]	2.55 (0.72 – 9.73)	0.66	0.156
	(38.2,104]	1.86 (0.53 – 6.84)	0.65	0.339
100	(26.6,37.2]	3.50 (0.98 – 13.75)	0.67	0.061
	(37.2,52.7]	1.91 (0.53 – 7.31)	0.66	0.330
	(52.7,103]	2.10 (0.57 – 8.16)	0.67	0.268
500	(80, 99.7]	1.83 (0.53 – 6.64)	0.64	0.344
	(99.7, 134]	1.35 (0.38 – 4.91)	0.65	0.643
	(134, 286]	1.00 (0.28 – 3.59)	0.64	1.000
**Area fraction**				
50	(5.8,9.8]	0.97 (0.27 – 3.43)	0.64	0.962
	(9.8,17.1]	1.20 (0.34 – 4.24)	0.64	0.775
	(17.1,38.5]	1.33 (0.39 – 4.65)	0.63	0.647
100	(6.7,11.4]	0.92 (0.26 – 3.17)	0.63	0.890
	(11.4,16.6]	1.22 (0.34 – 4.46)	0.65	0.758
	(16.6,36.9]	1.00 (0.28 – 3.51)	0.64	1.000
500	(7.9,12.4]	0.75 (0.21 – 2.57)	0.63	0.647
	(12.4,17.3]	1.25 (0.35 – 4.57)	0.62	0.732
	(17.3,37]	0.54 (0.15 – 1.90)	0.65	0.339
**Total area**				
50	(355,614]	2.85 (0.80 – 11.03)	0.66	0.114
	(614,1.19e + 03]	2.10 (0.57 – 8.16)	0.67	0.268
	(1.19e + 03,2.96e + 03]	2.33 (0.65 – 8.95)	0.66	0.201
100	(2.12e + 03,3.57e + 03]	1.00 (0.28 – 3.51)	0.64	1.000
	(3.57e + 03,5.15e + 03]	1.10 (0.31 – 3.93)	0.64	0.882
	(5.15e + 03,1.15e + 04]	1.00 (0.28 – 3.51)	0.64	1.000
500	(5.97e + 04,9.36e + 04]	1.00 (0.28 – 3.51)	0.64	1.000
	(9.36e + 04,1.28e + 05]	1.34 (0.38 – 4.89)	0.64	0.634
	(1.28e + 05,2.79e + 05]	0.81 (0.23 – 2.87)	0.64	0.749
**Count**				
50	(16,28]	0.38 (0.10 – 1.28)	0.64	0.125
	(28,38]	0.41 (0.10 – 1.49)	0.67	0.184
	(38,76]	0.92 (0.26 – 3.17)	0.63	0.890
100	(61,81]	0.36 (0.10 – 4.26)	0.65	0.117
	(81,146]	0.28 (0.07 – 1.02)	0.67	0.059
	(146,269]	0.62 (0.17 – 2.12)	0.63	0.444
500	(704,881]	0.82 (0.23 – 5.84)	0.63	0.752
	(881,1.03e + 03]	0.73 (0.20 – 2.57)	0.64	0.621
	(1.03e + 03,1.42e + 03]	0.82 (0.23 – 2.84)	0.63	0.752

## Conclusions

ImageJ provides a novel, free, and easy way to evaluate vegetation around homes for use in epidemiological analysis. This method eliminates the need for complex calculations to quantify vegetation (i.e. NDVI), as the output from the program provides raw numbers that can be imported directly into statistical software for analysis. Additionally, it standardizes how these images are analysed, which removes measurement error introduced when analysing by hand.

The methods developed here use only some of the many capabilities of ImageJ. Further experimentation on a wider range of landscapes and image types will enable implementation of even more practical uses of this freely available software. In this analysis, images were required to be in 8 bit black and white, potentially causing loss of important features. Recent methods have been developed using ImageJ for cell staining to recognize and select areas by colour [14]. This could be beneficial when analysing satellite data, to ensure unwanted features such as bodies of water, livestock, or houses, are not included in the analysis. Specifically important for malaria epidemiology would be a way to select areas based on size; as grasses and large trees do not typically provide suitable shelter for mosquitoes, excluding them from the analysis is important to ensure accuracy when modeling. While this was not evaluated in depth in this paper, ImageJ does have the capacity to select areas based on particle size.

There are many factors to be considered when studying malaria transmission, particularly in hypoendemic regions. The introduction of satellite data to epidemiological analysis has played an important role in advancing knowledge of how the environment affects malaria transmission, but these analyses are still complex and expensive, limiting the number of people who can put it to use. Using ImageJ is an important new tool to enhance satellite imagery analysis, as it is versatile, inexpensive, and provides quantitative output for use in epidemiological modelling.

## Competing interests

The authors declare that they have no competing interests.

## Authors’ contributions

ER conceptualized the study, analysed the data, performed statistical analysis, and wrote the manuscript. SF processed images, designed the macros required to run the analysis in Image J, and assisted with manuscript writing. CC participated in data collection. TL assisted with statistical analysis. CS assisted with study conceptualization and manuscript writing and revision. All authors read and approved the final manuscript.

## Supplementary Material

Additional file 1 **Image processing step 1.** Images must be converted to 8bit black and white images for analysis. This step will convert the colored image into a grey-scaled one. Click here for file

Additional file 2 **Image processing step 2.** Brightness and contrast must be set for an image. This allows separation of the plants from the background. The dark spots are the plants that will be counted. Click here for file

Additional file 3 **Image processing step 3.** A threshold must be set for the image. This removes all grayscale and calls any particle that is darker than the threshold black, and everything else white. Here, the negative was taken so that the white will be analysed. This is helpful for masking color images after the analysis (Additional file 8: Figure S8). Click here for file

Additional file 4 **Image processing step 4.** The image gets “despeckled” to remove all small background particles that made it through thresholding. In this example it was important to remove the buffer lines from the original image. Particles can then be analysed using ImageJ’s “Analyze Particles” function. This counts each white area and measures the size, then calculates the total area covered by white and the area fraction of this. These numbers can be summarized into averages for each category. This summary data was used in the statistical analysis. Click here for file

Additional file 5 **Image processing step 5.** After the threshold is set and the analysis run, the while particles, also called “masks,” can be pasted over the original image to check for accuracy. If too little or too much was covered by the masks, the threshold can be adjusted and the image re-analysed. Once a satisfactory threshold has been found this number is applied to the rest of the images for consistency. Click here for file

Additional file 6 Homestead 20, low resolution image (10 m/pixel), threshold 60. Click here for file

Additional file 7 Homestead 20, low resolution image (10 m/pixel), threshold 70. Click here for file

Additional file 8 Homestead 20, low resolution image (10 m/pixel), threshold 80. Click here for file

Additional file 9 Homestead 20, low resolution image (10 m/pixel), threshold 90. Click here for file

Additional file 10 Homestead 20, low resolution image (10 m/pixel), threshold 100. Click here for file
