# Supplementary figures and images for: Evaluating local vegetation cover as a risk factor for malaria transmission: a new analytical approach using ImageJ
Source: Malar J. 2014 Mar 13;13:94. doi: 10.1186/1475-2875-13-94 (PMC4007634; doi:10.1186/1475-2875-13-94)

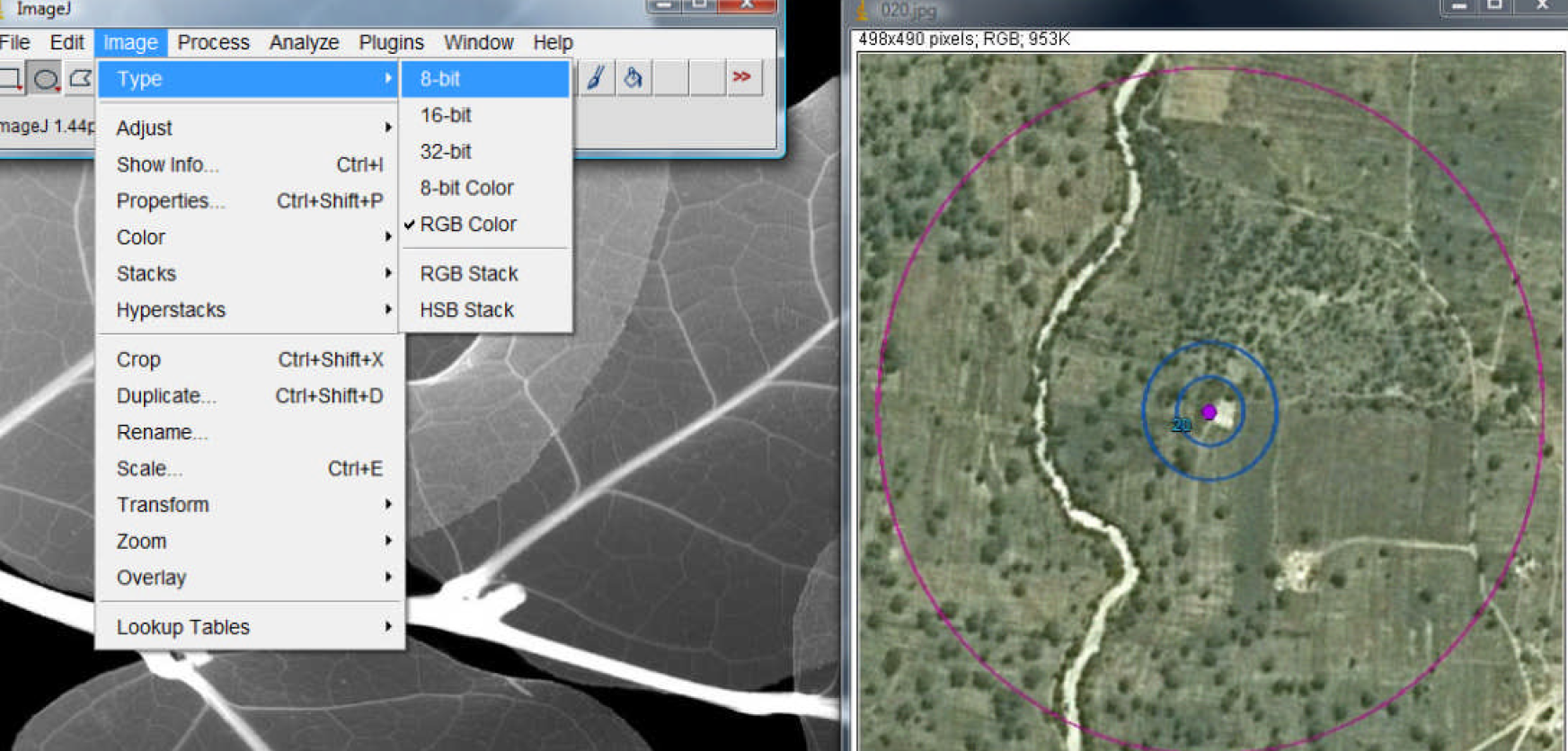

Supplement: Additional file 1 — Image processing step 1. Images must be converted to 8bit black and white images for analysis. This step will convert the colored image into a grey-scaled one. [file 1475-2875-13-94-S1.tiff]

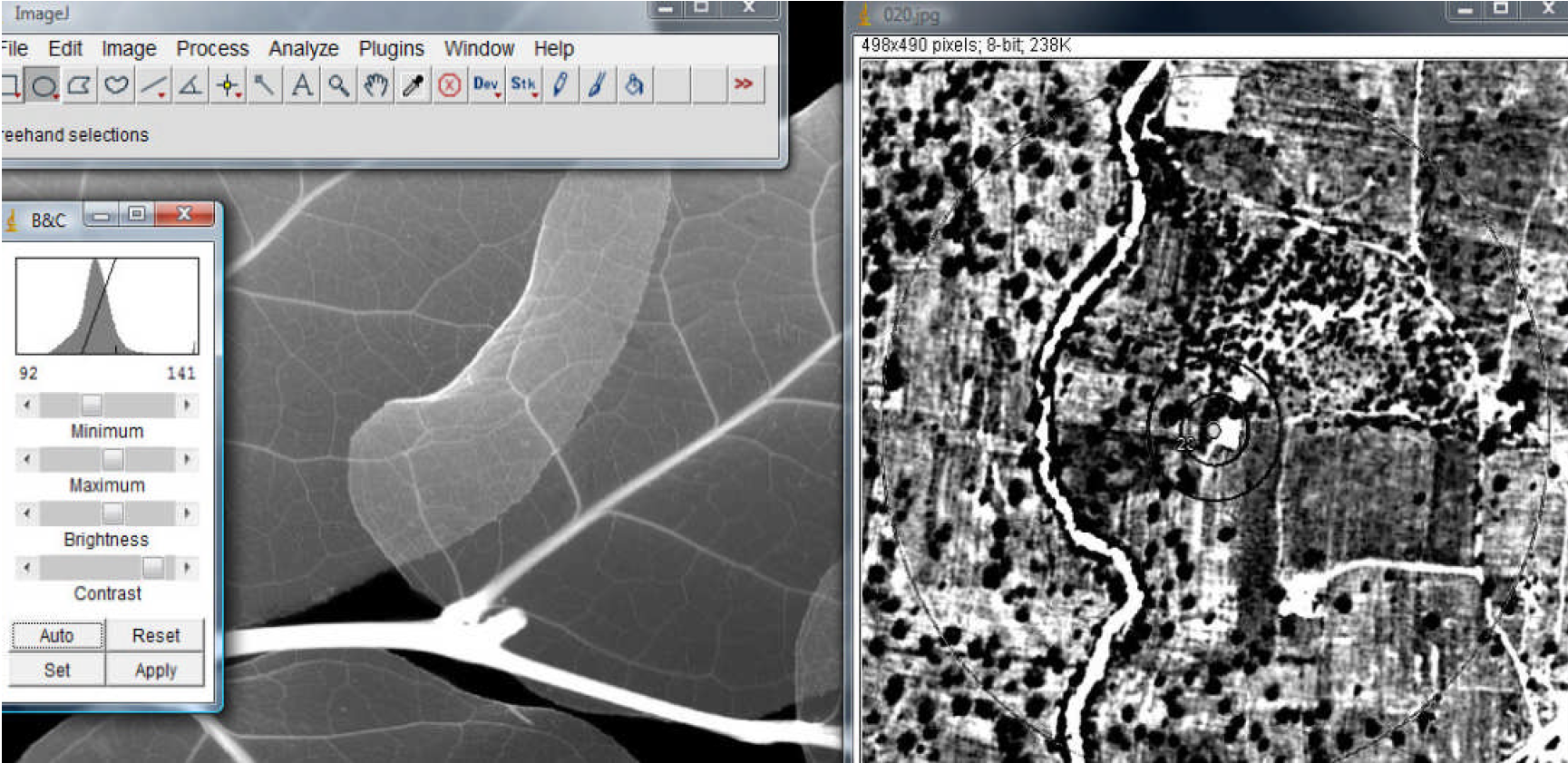

Supplement: Additional file 2 — Image processing step 2. Brightness and contrast must be set for an image. This allows separation of the plants from the background. The dark spots are the plants that will be counted. [file 1475-2875-13-94-S2.tiff]

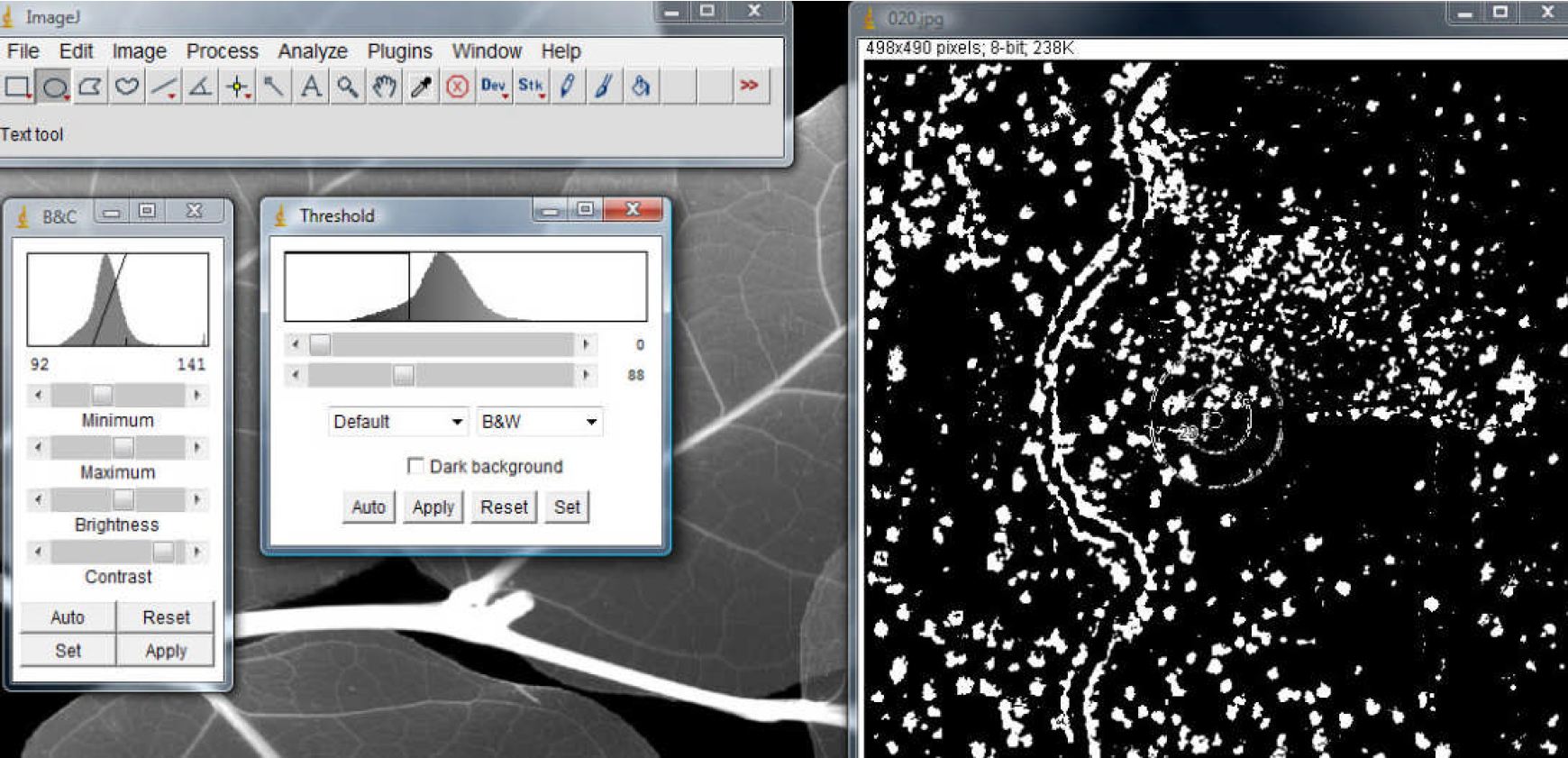

Supplement: Additional file 3 — Image processing step 3. A threshold must be set for the image. This removes all grayscale and calls any particle that is darker than the threshold black, and everything else white. Here, the negative was taken so that the white will be analysed. This is helpful for masking color images after the analysis (Additional file 8: Figure S8). [file 1475-2875-13-94-S3.tiff]

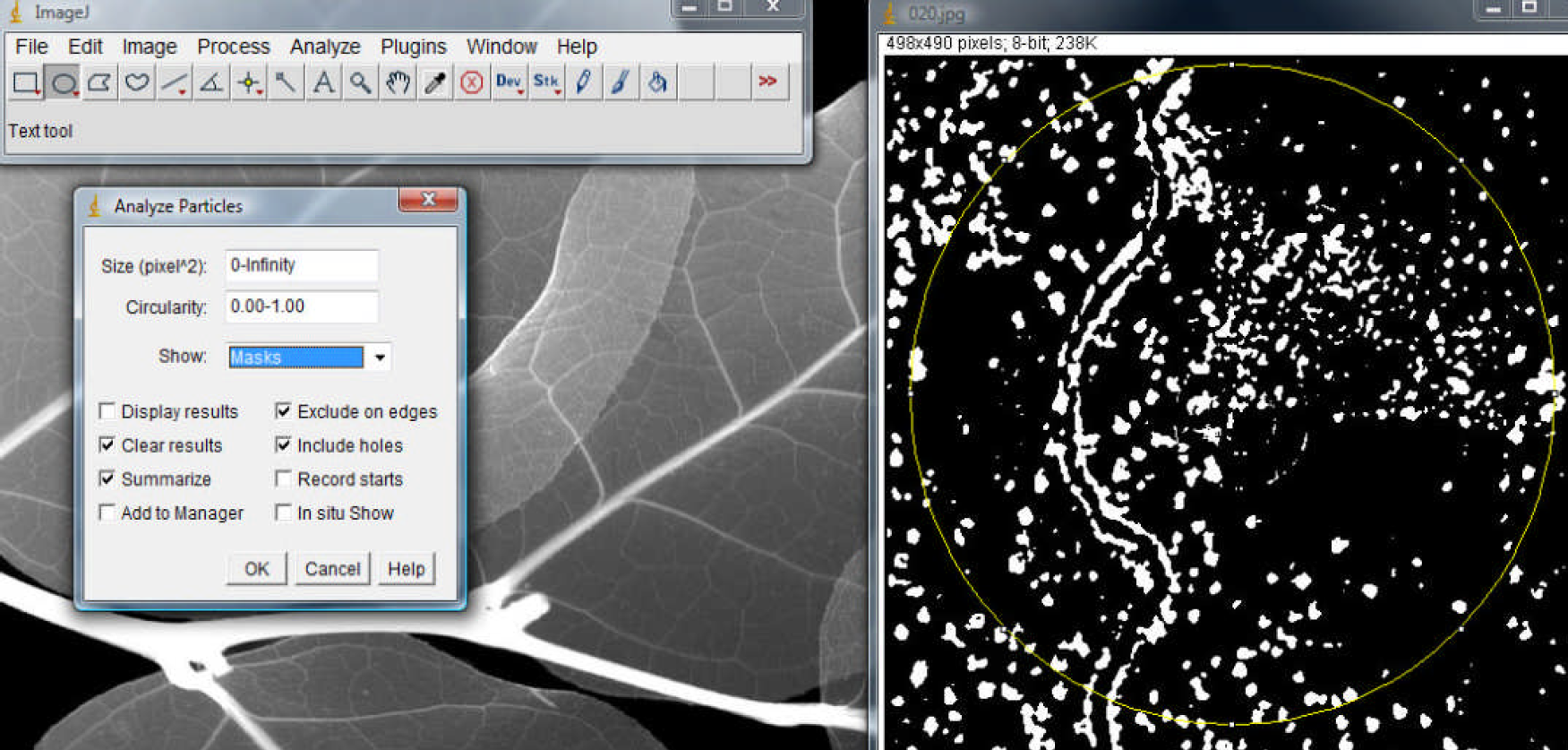

Supplement: Additional file 4 — Image processing step 4. The image gets “despeckled” to remove all small background particles that made it through thresholding. In this example it was important to remove the buffer lines from the original image. Particles can then be analysed using ImageJ’s “Analyze Particles” function. This counts each white area and measures the size, then calculates the total area covered by white and the area fraction of this. These numbers can be summarized into averages for each category. This summary data was used in the statistical analysis. [file 1475-2875-13-94-S4.tiff]

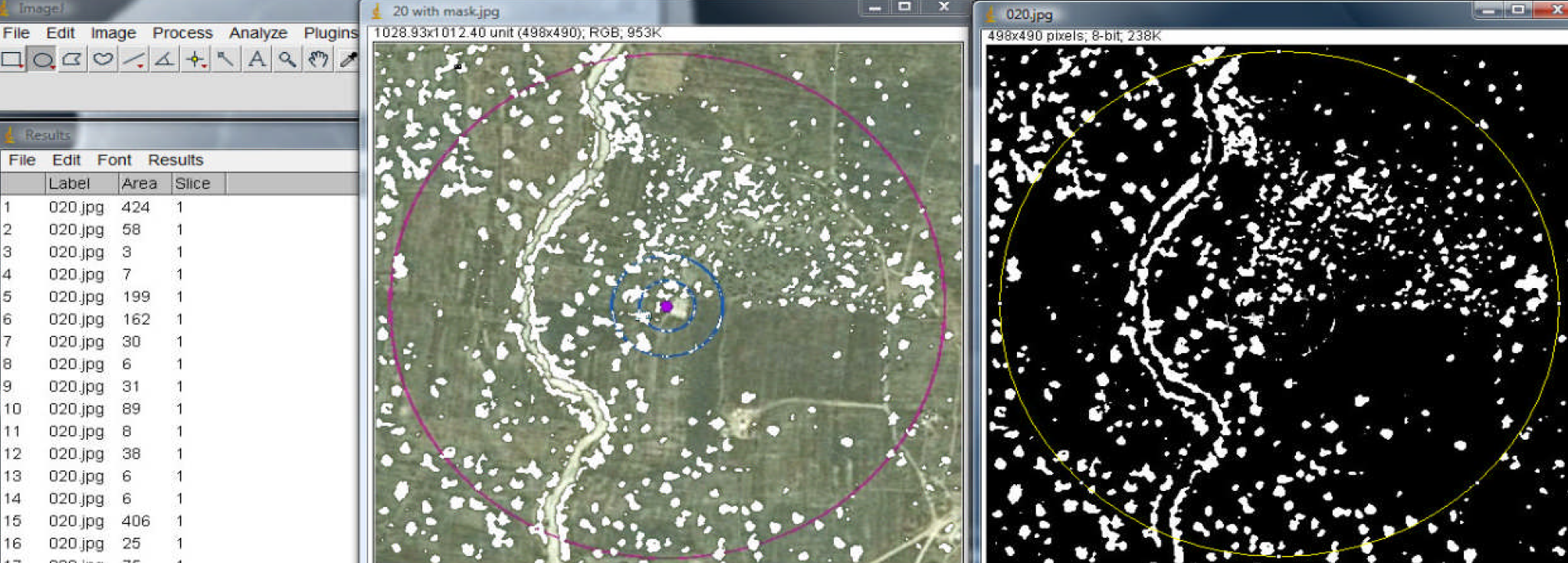

Supplement: Additional file 5 — Image processing step 5. After the threshold is set and the analysis run, the while particles, also called “masks,” can be pasted over the original image to check for accuracy. If too little or too much was covered by the masks, the threshold can be adjusted and the image re-analysed. Once a satisfactory threshold has been found this number is applied to the rest of the images for consistency. [file 1475-2875-13-94-S5.tiff]

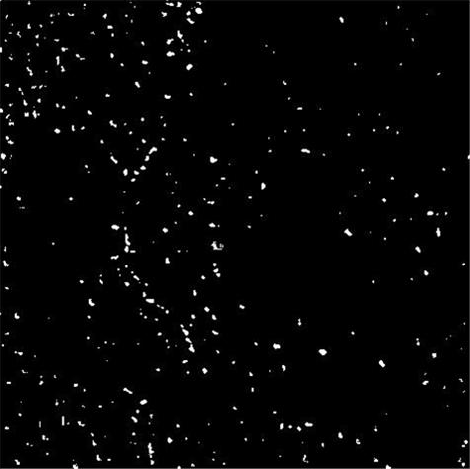

Supplement: Additional file 6 — Homestead 20, low resolution image (10 m/pixel), threshold 60. [file 1475-2875-13-94-S6.tiff]

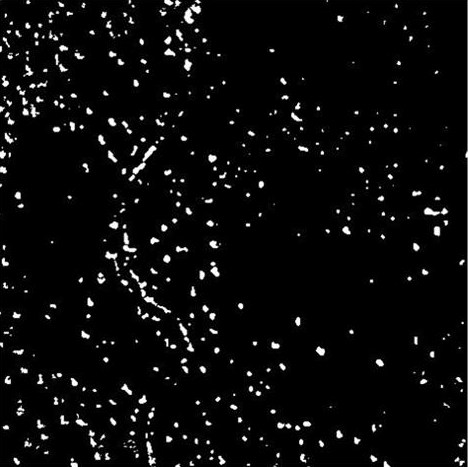

Supplement: Additional file 7 — Homestead 20, low resolution image (10 m/pixel), threshold 70. [file 1475-2875-13-94-S7.tiff]

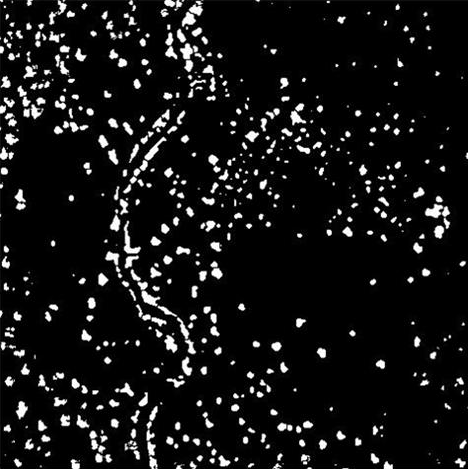

Supplement: Additional file 8 — Homestead 20, low resolution image (10 m/pixel), threshold 80. [file 1475-2875-13-94-S8.tiff]

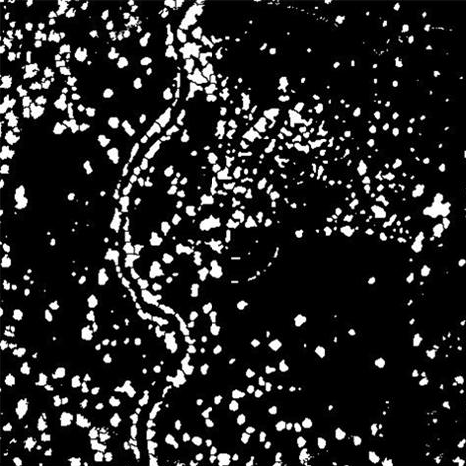

Supplement: Additional file 9 — Homestead 20, low resolution image (10 m/pixel), threshold 90. [file 1475-2875-13-94-S9.tiff]

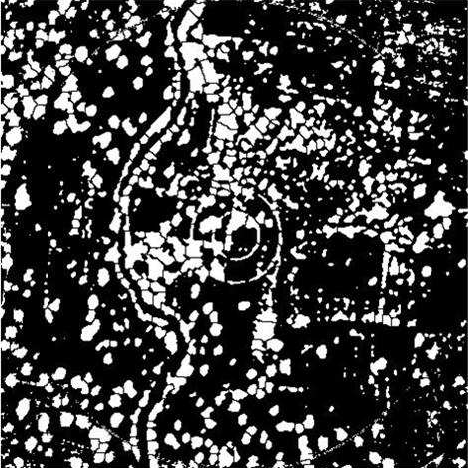

Supplement: Additional file 10 — Homestead 20, low resolution image (10 m/pixel), threshold 100. [file 1475-2875-13-94-S10.tiff]
